# Psychomotor and neurofunctional sequelae after COVID-19

**DOI:** 10.1017/neu.2026.10067

**Published:** 2026-03-05

**Authors:** Lara L.W. Chiminazzo, Ivana B. Suffredini, Thiago B. Kirsten

**Affiliations:** Psychoneuroimmunology Laboratory, Program in Environmental and Experimental Pathology, Paulista University, Brazil

**Keywords:** SARS-CoV-2, psychomotor disorders, coronavirus, pandemics, nervous system diseases

## Abstract

**Objective:**

A previous study by our research group identified psychomotor and neurofunctional impairments following SARS-CoV-2 infection. This study continues that investigation, aiming to evaluate whether these impairments persisted over time, as part of the broader characterisation of long COVID. Moreover, it was explored potential correlations with variables such as age, blood type, symptoms, and medical care.

**Methods:**

From an initial pool of 214 subjects, 30 post-COVID-19 participants and 30 healthy controls were selected after strict exclusion criteria. The assessments protocol included eight psychomotor tests – Fine Motor Development (Diadochokinesia, Puppets, Fan, and Paper) and Balance (Immobility, Static Balance on One Foot, Feet in Line, and Persistence) – as well as three cognitive screening tasks from the Mini-Mental State Examination: Episodic Memory After Distracters, Verbal Fluency, and Clock tests. Evaluations were performed at three time points: baseline (post-COVID-19), 12 weeks, and 24 weeks. Participants were stratified by age (18–30, 31–45, and 46–64 years), symptoms profile, medical care, and blood type.

**Results:**

COVID-19 induced psychomotor and neurofunctional sequelae lasting at least 24 weeks post-infection. These impairments were more pronounced and persistent in the 31–45-years age group, while memory-related impairments were more evident in the 18–30 age group. Body pain, coryza, and sore throat were key symptoms linked to long-term sequelae. Rh-negative blood type was suggested as a potential risk factor.

**Conclusion:**

The findings support that long COVID included sustained psychomotor and neurofunctional sequelae, premature senescence, and associations with specific clinical and biological variables.

Significant outcomes

- COVID-19 induced psychomotor and neurofunctional sequelae lasting at least 24 weeks after the acute phase of the disease. The impairments included deficits in fine motor development, balance, and Mini-Mental State Examination performance, as well as the induction of synkinesis.
- Different age groups showed distinct outcomes: psychomotor impairments were more pronounced and persisted longer in the 31–45 age group, whereas individuals aged 18–30 years exhibited greater memory impairments.
- Body pain, coryza, and sore throat during the acute infection were identified as key symptoms associated with psychomotor and neurofunctional sequelae. Additionally, Rh-negative blood type was suggested as a risk factor for COVID-19.

Limitations

- A major challenge was adapting long-established face-to-face tests to an online format due to pandemic restrictions. Connection issues and image–sound mismatches were significant problems. As a result, some key tests – such as the Tonicity Test and the Attention and Concentration Test (M. Stambak), which could clarify the influence of tonic state on attention – had to be excluded. For psychometricians, tonic dialogue with participants is essential to build trust and foster acceptance of difficulties; achieving this subtle interaction online required extra time for informal conversations to ease anxiety related to isolation and fear of the disease.
- Lack of brain imaging exams to compare and explain psychomotor outcomes.

## Introduction

Coronavirus Disease 2019 (COVID-19), caused by the Severe Acute Respiratory Syndrome Coronavirus 2 (SARS-CoV-2), has led to a global pandemic, resulting in over seven million deaths worldwide as of July 2025 (WHO, 2025).

Since the first reports of cases and symptoms, neurological manifestation were noted alongside the classic respiratory signs – such as fever, cough, coryza, loss of smell, breathing difficulties, and chest pain (Guan *et al*., 2020). For instance, in Wuhan, China, early observations in 2020 showed that 36–45% of COVID-19 patients presented neurological symptoms (Li *et al*., 2020).

Since then, several studies have confirmed a correlation between COVID-19 and cognitive impairments, including dizziness, myalgia, sleep disturbances, migraine, headaches, dysgeusia, ageusia, hyposmia, anosmia, neurocognitive deficits, encephalopathy, encephalitis, disorientation, poorly organised motor responses, Guillain-Barre syndrome, and stroke (Doskas *et al*., 2025; Fanshawe *et al*., 2025), as well as neuropsychiatric disorders such as depression, psychosis, and anxiety (Yousufzai *et al*., 2025). It is estimated that up to one-third of COVID-19 survivors experience at least one neurological complication (Misra *et al*., 2021), with symptoms persisting for more than two years after diagnosis (Yousufzai *et al*., 2025).

COVID-19 appears to affect the central nervous system directly and/or indirectly, inducing neurological disorders (Li *et al*., 2020; Doskas *et al*., 2025). The underlying neurobiological mechanisms remain unclear but may involve the virus’s neuroinvasive and neurotropic properties, immune-mediated responses, and systemic inflammation (Wu *et al*., 2020; Borczuk and Yantiss, 2022; Yousufzai *et al*., 2025). These processes can cause persistent neuronal damage, disrupting synaptic signalling of upper-layer excitatory neurones, similar to changes observed in neurodegenerative diseases and early senescence (Borczuk & Yantiss, 2022). Indeed, a detailed domain-specific phenotype of the cognitive impairments associated with so-called long COVID has yet to be fully characterised (Fanshawe *et al*., 2025).

Long COVID is expected to cause substantial global morbidity for many years, with worldwide infections surpassing 500 million and a conservative prevalence estimate of 20–30% (Byambasuren *et al*., 2023). Given its wide range of symptoms, relapsing/remitting nature, and diverse underlying mechanisms, diagnosis and classification remain challenging (Fernandez-de-Las-Penas *et al*., 2021), underscoring the need for further studies. Moreover, there is limited mass-media coverage emphasising the importance of seeking clinical support after initial COVID-19 symptoms resolve, when new symptoms appear, or when symptoms persist beyond 24 weeks post-diagnosis (Mahase, 2020).

During the pandemic, our research group developed and applied tests to assess psychomotor and neurofunctional outcomes after recovery from the acute phase of COVID-19 (Chiminazzo & Kirsten, 2023). Results revealed impairments in Fine Motor Development, Balance, Memory, Verbal Fluency, Clock, and Synkinesis tests compared with control data. These findings are striking considering that most participants reported no pre-existing conditions and did not require hospitalisation. The present study extends this project to evaluate long COVID by monitoring these subjects at 12 and 24 weeks after the first evaluation, to determine whether psychomotor and neurofunctional impairments were transient or persistent. It was also objective to explore correlations between the sequelae and variables such as age and blood type, as well as to identify key symptoms and the level of medical care during the acute phase associated with later deficits. Notably, the 12- and 24-week assessments align with the minimum and maximal timeframes commonly used to define long post-COVID symptoms (Fernandez-de-Las-Penas *et al*., 2021).

## Material and methods

### Ethical standards

The authors assert that all procedures contributing to this work comply with the ethical standards of the relevant national and institutional committees on human experimentation and with the Helsinki Declaration of 1975, as revised in 2008. This project was approved by the Research Ethics Committee of the Paulista University (CEP-UNIP no. 34780720.4.0000.5512). All participants in this study took part voluntarily and only after providing informed consent, in accordance with the free and informed consent form (Chiminazzo & Kirsten, 2023).

### Experimental protocols, research subjects, and experimental design

The experimental protocols were designed, developed, and tested by our research group (Chiminazzo & Kirsten, 2023), and are described in detail in the *Manual for Psychomotor and Neurofunctional Assessment after COVID-19* (Chiminazzo & Kirsten, 2024). The tests were adapted from studies of Rossel (1975); Guilmain and Guilmain (1986); Brêtas (1991) and Nitrini and colleagues (1994).

A total of 214 subjects were evaluated using convenience sample: 184 post-COVID-19 subjects and 30 healthy volunteers (control group, with no history of flu-like symptoms in the three months prior to the study). The inclusion and exclusion criteria, as well as the epidemiological data (Phase 1), were previously described (Chiminazzo & Kirsten, 2023).

From Phase 1, individuals were selected for Phase 2 based on the following exclusion criteria: (a) age (under 18 or over 64 years); (b) no confirmed positive test for COVID-19, (c) time since diagnosis (less than 15 days or more than seven months after the positive COVID-19 diagnosis); (d) present flu-like symptoms on the day of evaluation; and (e) absence of at least three of the following symptoms of interest–loss/alteration of smell and/or taste, dizziness, nausea and/or vomiting, ataxia, headaches, difficulty breathing, chest pain/pressure, and speech impairment (Brito & Silva, 2020).

For the Phase 2, 30 subjects from the control group and 30 subjects from the post-COVID-19 group were randomly selected based on the exclusion criteria and the refusal of some individuals to participate. Participants in both groups were also selected to ensure an equal age distribution across groups (18–30, 31–45, and 46–64 years).

In Phase 2, control and post-COVID-19 participants were evaluated for psychomotor and neurofunctional aspects, including eight psychomotor tests and three Mini-Mental State Examination (MMSE) tests (Chiminazzo & Kirsten, 2023). The eight psychomotor tests were: Fine Motor Development Test – Diadochokinesia, Fine Motor Development Test – Puppets, Fine Motor Development Test – Fan, Fine Motor Development Test – Paper, Balance Test – Immobility, Balance Test – Static Balance on One Foot, Balance Test – Feet in Line, and Balance Test – Persistence. The three MMSE tests were: Episodic Memory After Distracters, Verbal Fluency, and Clock. Detailed descriptions of each test, their applications, and specific procedures have been reported previously (Chiminazzo & Kirsten, 2024).

Subjects in Phase 2 were evaluated at three different time points using the same psychomotor and neurofunctional assessments: Evaluation 1 – baseline; Evaluation 2 – 12 weeks after the Evaluation 1; and Evaluation 3 – 24 weeks after the Evaluation 1.

### Statistical analysis

For statistical purposes, the results of the Fine Motor Development Tests (Diadochokinesia, Puppets, Fan, and Paper) and the Balance Tests (Immobility, Static Balance on One Foot, Feet in Line, and Persistence) were combined to represent the overall performance on the psychomotor tests, which encompasses motor and balance responses (Chiminazzo & Kirsten, 2023). The results of this combined measure are referred to as ‘Psychomotor Tests’. The Episodic Memory After Distracters Test is referred to as the ‘Memory Test’.

One of the purposes of the Fine Motor Development Puppets and Fan Tests is to evaluate the presence of synkinesis (i.e., involuntary or parasitic movements); therefore, ‘Synkinesis’ was also included as an evaluated parameter.

Normality of the data distribution was assessed using the Shapiro–Wilk (W) or Kolmogorov–Smirnov (KS) tests, depending on sample size. Bartlett’s test was used to evaluate homoscedasticity. When necessary, an outlier detection test (ROUT, *Q* = 5%) was applied. One-way analysis of variance (ANOVA) followed by Tukey’s multiple-comparison post hoc test was used for parametric data, while the Kruskal–Wallis test followed by Dunn’s post hoc test was applied for nonparametric data. Spearman’s correlation coefficient was calculated to examine positive and negative correlations between multiple variables in Evaluation 1. The Spearman coefficient ranges from −1 to 1, where a positive value indicates that two variables move in the same direction and a negative value indicates that they move in opposite directions (i.e., as one increases, the other decreases). Values approaching either extreme (±1) were considered potentially biologically relevant (Schober *et al*., 2018). Results were considered significant at *p* < 0.05.

Data are expressed as mean ± SEM and are displayed as box-and-whiskers (min to max, showing all values) or violin plots. Correlation results are also presented as heat maps based on Spearman’s coefficients: warm colours (yellow to red) progressively indicated positive correlations, whereas cool colours (medium blue to lilac) progressively indicated negative correlations.

All statistical tests and graphic illustrations were performed using GraphPad Prism (v. 8.0.2, GraphPad Software, San Diego, USA).

## Results

The initial results obtained after applying the inclusion and exclusion criteria, as well as the epidemiological data (Phase 1), are presented in a previous study (Chiminazzo & Kirsten, 2023). These data include the distribution by gender and age, the period of COVID-19 diagnosis, clinical symptoms, pre-existing diseases, level of medical care, health and daily routine after COVID-19, type of COVID-19 testing, and blood type. For Phase 2 – Evaluation 1, in both the control and post-COVID-19 groups (*n* = 30/group), there were six participants aged 18–30 years (20%), 13 participants aged 31–45 years (43,33%), and 11 participants aged 46–64 years(36,66%) (Chiminazzo & Kirsten, 2023).

Participant attrition occurred between Evaluations 2 and 3. In the Phase 2 – Evaluation 2 (after 12 weeks), ten participants discontinued from the control group: two withdrew (i.e., did not respond to the study’s communication channels), and eight were excluded due to being diagnosed with COVID-19. Thus, for Evaluation 2, the sample size was 20 for the control group and 30 for the post-COVID-19 group. In the Phase 2 – Evaluation 3 (after 24 weeks), seven participants discontinued from the control group (due to a COVID-19 diagnosis), and two participants discontinued from the post-COVID-19 group (due to initiating psychiatric treatment and psychotropic medication – initial exclusion criteria for the study). Therefore, for Evaluation 3, the sample size was 13 for the control group and 28 for the post-COVID-19 group.

### Persistence of psychomotor and neurofunctional impairments

**Figure 1.**
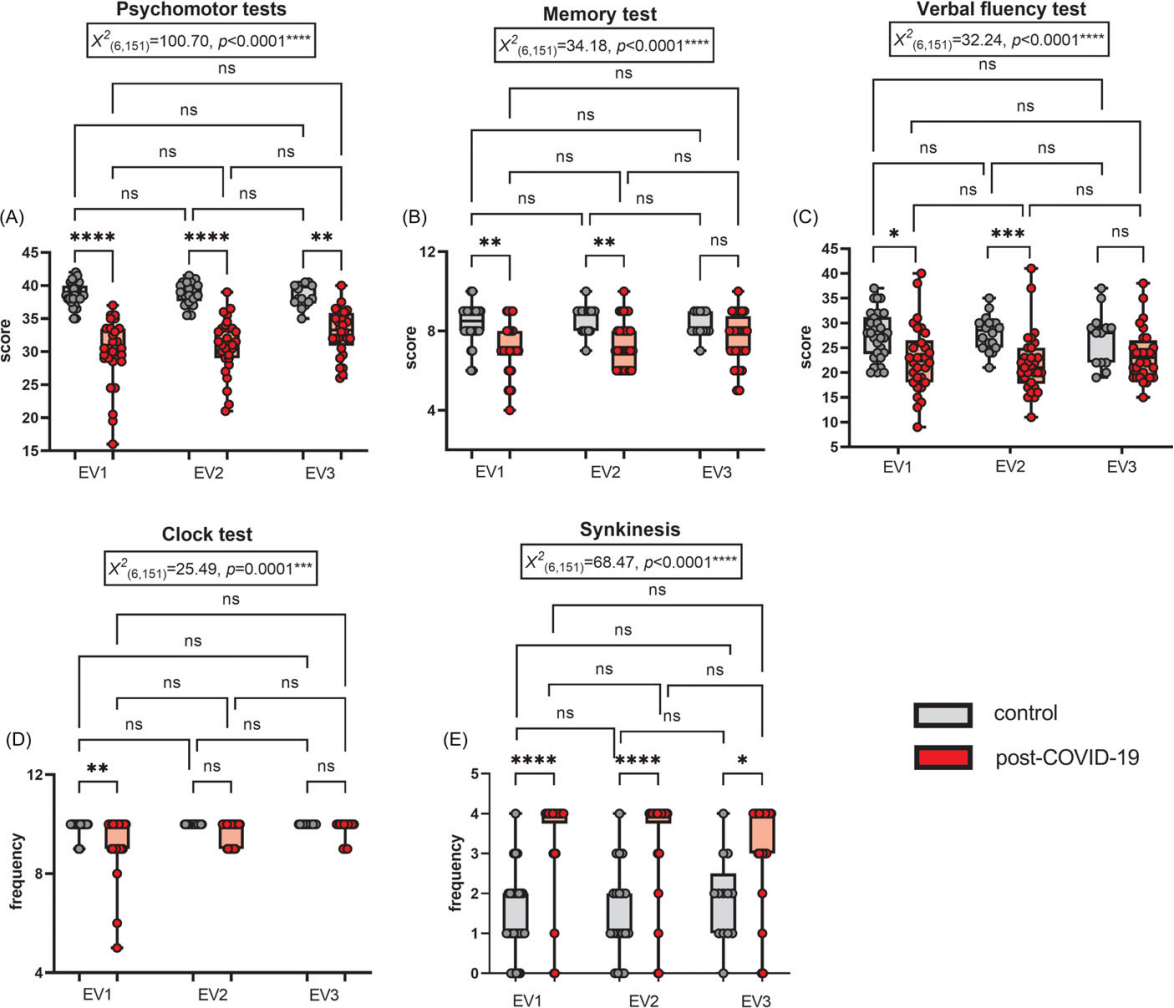
Performance in the (A) Psychomotor tests (including fine motor development and balance assessments), (B) Memory test, (C) Verbal fluency test, (D) Clock test, and (E) Synkinesis frequency assessed on phase 2 – evaluations (EV) 1 (initial), 2 (after 12 weeks), and 3 (after 24 weeks). All assessments included comparisons of the post-COVID-19 group with the respective control groups (*n* = 30/30 on EV1, 20/30 on EV2, and 13/28 on EV3 for the control and post-COVID-19 groups, respectively; Kruskal–Wallis test followed by Dunn’s post-test). *p < 0.05, **p < 0.01, ***p < 0.001, and ****p < 0.0001. The results are expressed as mean ± SEM and with box & whiskers (min to max, showing all values) graphs.

The post-COVID-19 group demonstrated impairments in all three Evaluations of the Psychomotor Tests, which included assessments of Fine Motor Development (Diadochokinesia, Puppets, Fan, and Paper) and Balance (Immobility, Static Balance on One Foot, Feet in Line, and Persistence), when compared to the control group (Figure 1A). Thus, even 12 and 24 weeks after the initial assessment, COVID-19 continued to be associated with significant psychomotor impairments.

Memory and Verbal Fluency tests revealed impairments in the post-COVID-19 group during Evaluations 1 and 2, respectively (Figure 1B and 1C). However, by Evaluation 3, performance in these domains was comparable to that of the control group.

For the Clock Test, the impairment initially observed in the post-COVID-19 group was no longer evident at 12 and 24 weeks (Figure 1D).

The post-COVID-19 group showed a higher frequency of Synkinesis compared to the control groups at the three evaluation points (Figure 1E).

Supplementary Table 1 presents the results of the normality tests conducted for the Psychomotor Tests, Memory Test, Verbal Fluency Test, Clock Test, and Synkinesis Frequency.

In summary, the psychomotor and neurofunctional impairments identified in the initial post-COVID-19 assessment persisted after 12 and 24 weeks, particularly in Fine Motor Development and Balance tests, as well as in the continued high incidence of Synkinesis. Some impairments – specifically those related to Memory and Verbal Fluency – were alleviated only after 24 weeks from initial assessment.

### Psychomotor and neurofunctional impairments across age groups

**Figure 2.**
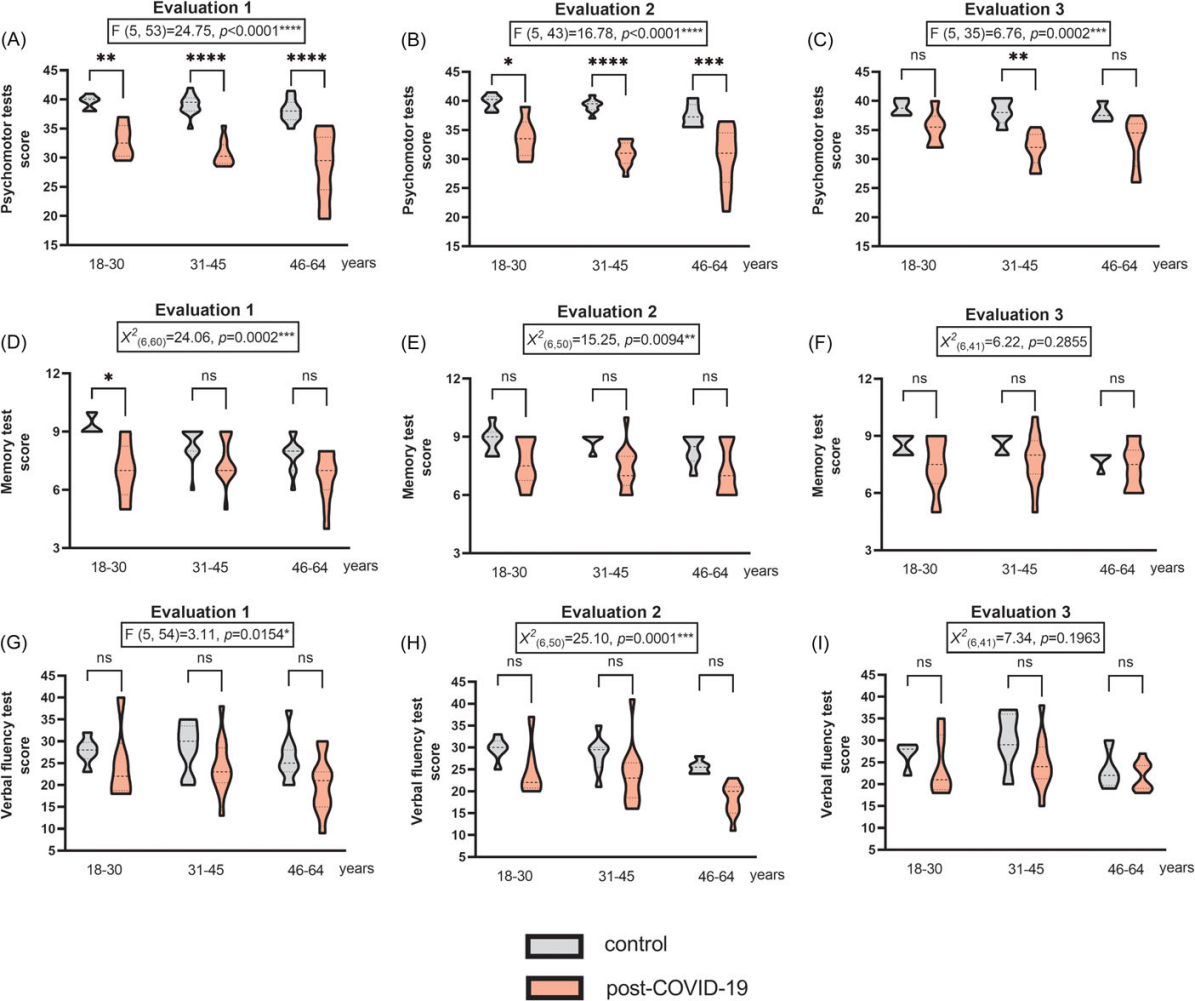
Performance by age in the (A–C) Psychomotor tests (including fine motor development and balance assessments), (D–F) Memory test, and (G–I) Verbal fluency test assessed on phase 2 – evaluations 1 (initial), 2 (after 12 weeks), and 3 (after 24 weeks). All assessments included comparisons of the post-COVID-19 group with the respective control groups (*n* = 30/30 on EV1, 20/30 on EV2, and 13/28 on EV3 for the control and post-COVID-19 groups, respectively; one-way ANOVA followed by Tukey’s post-test and Kruskal–Wallis test followed by Dunn’s post-test). *p < 0.05, **p < 0.01, ***p < 0.001, and ****p < 0.0001. The results are expressed as mean ± SEM and with violin plots.

The post-COVID-19 group showed impairments in the Psychomotor Tests across all three age groups in both Evaluation 1 (Figure 2A) and Evaluation 2 (Figure 2B). Notably, the impairment was most pronounced in the 31–45 age group, followed by the 46–64 and 18–30 age groups, always compared in comparison with their respective control groups. However, in Evaluation 3 (Figure 2C), only the 31–45 age group continued to show psychomotor impairment relative to its control group.

In the Memory Test, post-COVID-19 impairments were detected in Evaluation 1 (Figure 2D) and Evaluation 2 (Figure 2E). However, unlike the Psychomotor Tests, only the 18–30 age group showed impairment in Evaluation 1 compared with its control group. The 31–45 and 46–64 age groups did not show impairments in the Memory Test compared with their control groups in any of the three assessments. No differences were observed in Evaluation 3 (Figure 2F).

In the Verbal Fluency Test, post-COVID-19 impairments were also observed in Evaluation 1 (Figure 2G) and Evaluation 2 (Figure 2H), with no significant changes in Evaluation 3 (Figure 2I). However, none of the three age groups showed specific impairments in Verbal Fluency compared with their respective control groups in any of the three assessments.

Supplementary Table 2 presents the results of the normality tests performed for the Psychomotor Tests, Memory Test, and Verbal Fluency Test evaluated across the three age groups.

Thus, the different age groups responded differently to psychomotor and neurofunctional testing over time. Post-COVID-19 impairments in the Psychomotor Tests were more pronounced and longer-lasting in the 31–45 age group. In the Memory Test, post-COVID-19 impairments were more pronounced in the 18–30 age group.

### Relationship between COVID-19 symptoms and psychomotor/neurofunctional performance

**Figure 3.**
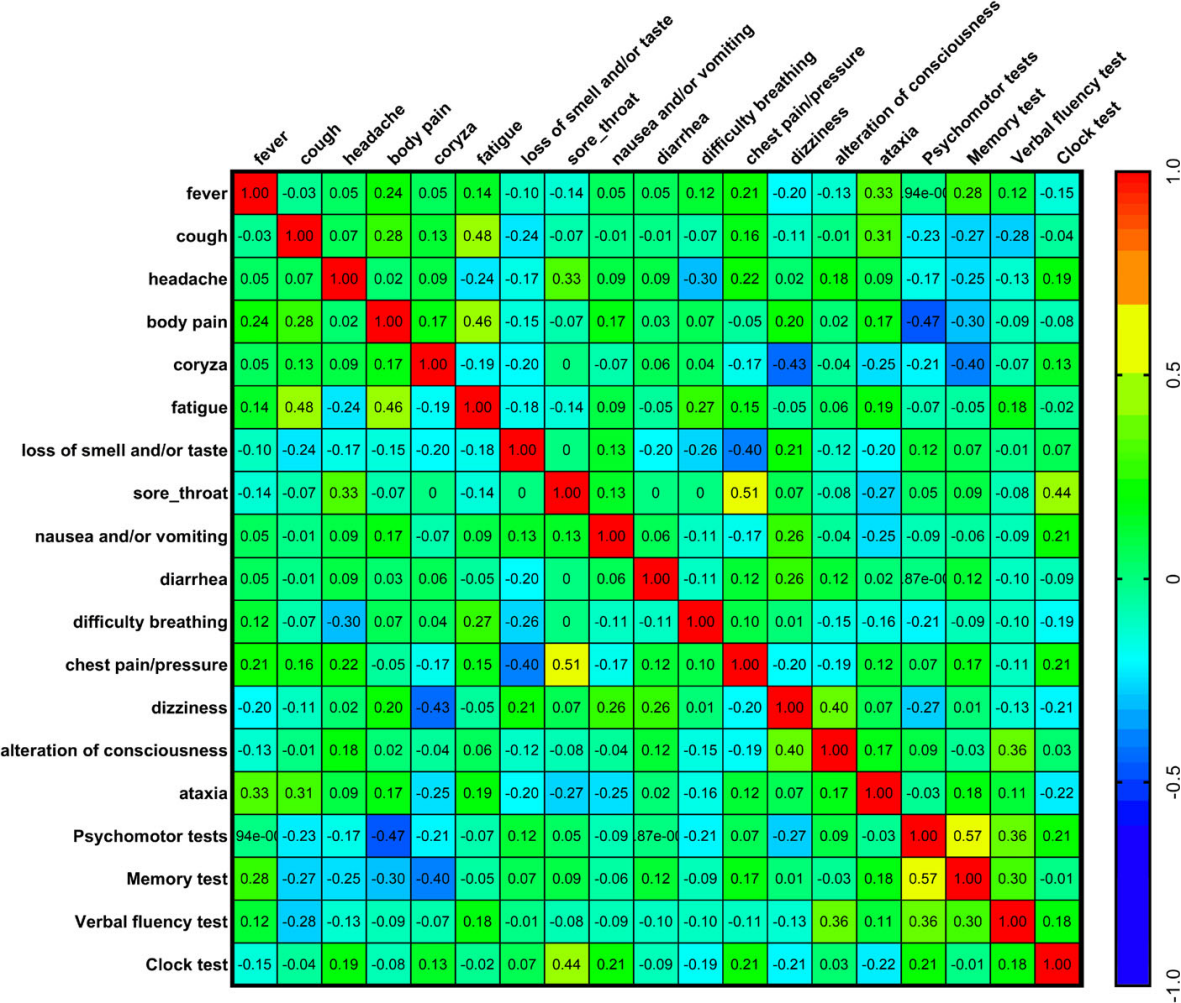
Comparisons between COVID-19 symptoms versus psychomotor and neurofunctional performance (Psychomotor, memory, verbal fluency, and clock tests) in evaluation 1 (*n* = 30, Spearman’s correlation coefficient test). Heat map: warm colours, between yellow and red, progressively revealed a positive correlation between two factors; and cool colours, between medium blue and lilac, progressively revealed a negative correlation between two factors.

When comparing each symptom reported during the course of COVID-19 with performance on psychomotor and neurofunctional tests, statistically significant correlations (Spearman’s correlation coefficient) were observed. Specifically, the following correlations were found: body pain versus Psychomotor Tests (*p* = 0.009**); coryza versus Memory Test (*p* = 0.030*); sore throat versus Clock Test (*p* = 0.015*); and alteration of consciousness versus Verbal Fluency Test (*p* = 0.047*). Figure 3 shows the heat map generated using Spearman’s correlation analysis. Negative correlations were detected for body pain versus Psychomotor Tests (*r* = −0.47) and coryza versus Memory Test (*r* = −0.40). In other words, the presence of symptoms such as body pain, coryza, sore throat, and alteration of consciousness was associated with poorer performance in post-COVID-19 psychomotor and neurofunctional tests. These symptoms were therefore considered key indicators of post-COVID-19 psychomotor and neurofunctional impairments. Other negative correlations between symptoms and test performance were also observed, but to a lesser extent and without statistical significance (Figure 3).

Significant correlations were also observed when comparing individual symptoms: cough versus fatigue (*p* = 0.008**); body pain versus fatigue (*p* = 0.010*); dizziness versus coryza (*p* = 0.016*); loss of smell and/or taste versus chest pain/pressure (*p* = 0.028*); sore throat versus chest pain/pressure (*p* = 0.004**); and dizziness versus alteration of consciousness (*p* = 0.029*). Figure 3 shows positive correlations for cough versus fatigue (*r* = 0.48); body pain versus fatigue (*r* = 0.46); sore throat versus chest pain/pressure (*r* = 0.51); and dizziness versus alteration of consciousness (*r* = 0.40). Negative correlations were observed for dizziness versus coryza (*r* = −0.43) and loss of smell and/or taste versus chest pain/pressure (*r* = −0.40). In other words, some symptoms tended to occur together, whereas others tended to occur in the absence or with lower incidence of each other.

Finally, a significant correlation was found between the Psychomotor and Memory tests (*p* = 0.001**). As shown in Figure 3, this association was positive (*r* = 0.57), indicating that post-COVID-19 patients who performed poorly on the Psychomotor Tests also tended to perform poorly on the Memory Test.

### Impact of medical care during COVID-19 on psychomotor and neurofunctional performance

**Figure 4.**
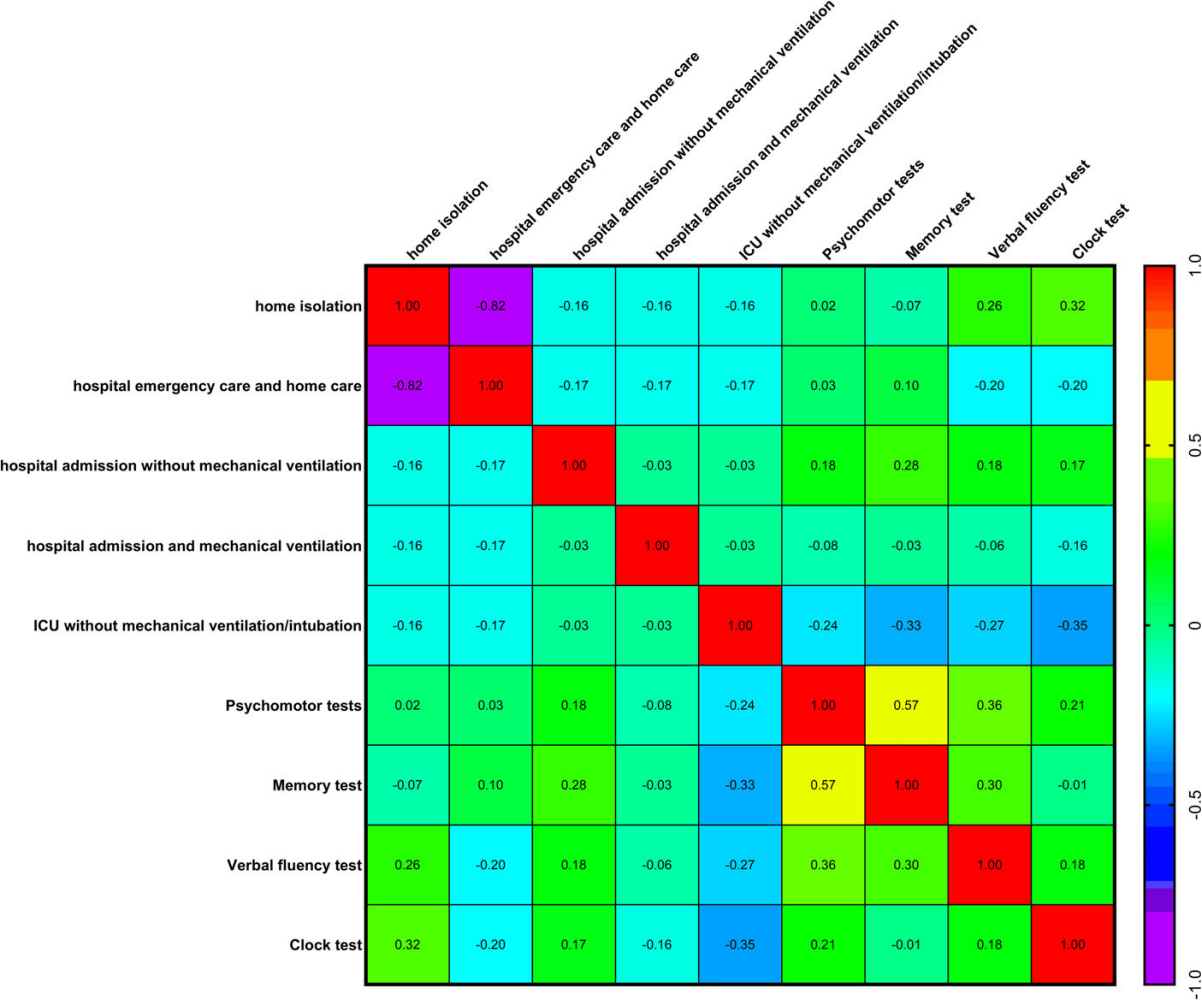
Comparisons between medical care during COVID-19 versus psychomotor and neurofunctional performance (Psychomotor, memory, verbal fluency, and clock tests) in evaluation 1 (*n* = 30, Spearman’s correlation coefficient test). Heat map: warm colours, between yellow and red, progressively revealed a positive correlation between two factors; and cool colours, between medium blue and lilac, progressively revealed a negative correlation between two factors.

During the course of COVID-19, patients required or chose different levels of medical care: no changes in routine; home isolation; hospital emergency care with subsequent home care; hospital admission without mechanical ventilation; hospital admission with mechanical ventilation; intensive care unit (ICU) admission without mechanical ventilation or intubation; and ICU admission with mechanical ventilation and/or intubation. When comparing each level of medical care with performance on psychomotor and neurofunctional tests, no significant correlations (Spearman’s correlation coefficient) or statistical differences were found. In other words, none of the levels of medical care was a determinant of better or worse performance on the psychomotor and neurofunctional tests (Figure 4). Thus, a patient who experienced severe COVID-19 could recover without psychomotor or neurofunctional sequelae, whereas a patient with only mild symptoms and no hospitalisation could still present marked psychomotor and neurofunctional impairments.

Only one significant (negative) correlation was observed when comparing each medical care levels: home isolation versus hospital emergency care (*r* = −0.82, *p* = 3.39047087681238e-008****, Figure 4).

As with the results for symptoms and psychomotor/neurofunctional performance, a significant positive correlation was also found between the Psychomotor versus Memory tests (*r* = 0.57, *p* = 0.001**, Figure 4).

### Influence of blood type and Rh factor on psychomotor and neurofunctional performance

**Figure 5.**
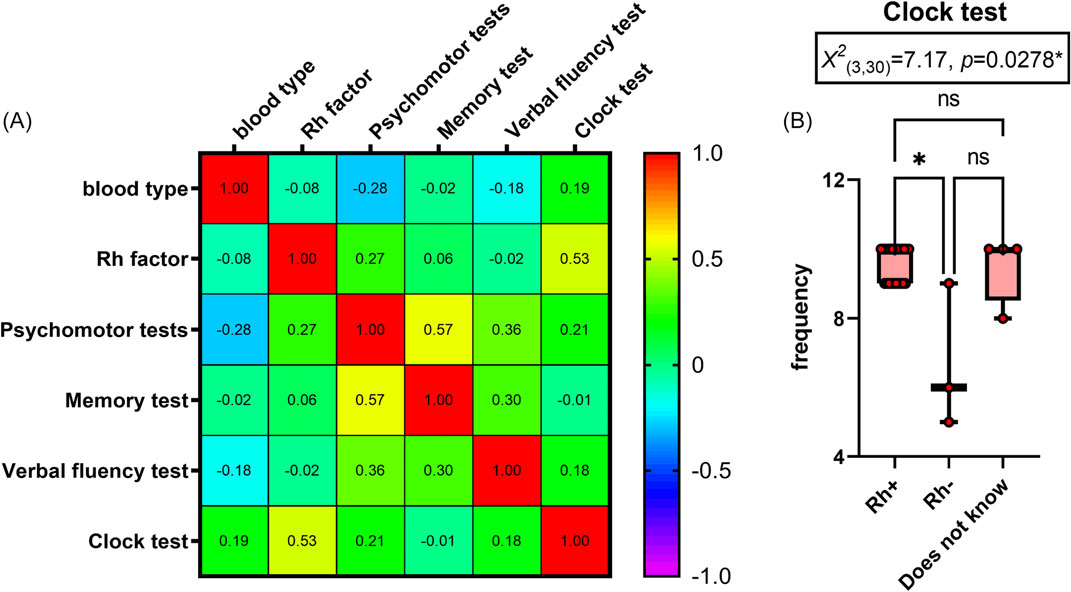
Comparisons between blood type and rh factor versus psychomotor and neurofunctional performance (Psychomotor, memory, verbal fluency, and clock tests) post-COVID-19 in evaluation 1 (*n* = 30, Spearman’s correlation coefficient test). (A) Heat map: warm colours, between yellow and red, progressively revealed a positive correlation between two factors; and cool colours, between medium blue and lilac, progressively revealed a negative correlation between two factors. (B) Kruskal–Wallis test followed by Dunn’s post-test. *p < 0.05; the results are expressed as mean ± SEM and with box & whiskers (min to max, showing all values) graphs.

Among the four blood types (A, B, AB, and O), it was investigated whether any were associated with differences in psychomotor and neurofunctional test performance, or whether they might offer a protective effect, for example. When comparing blood types with each psychomotor and neurofunctional test, no correlations (based on Spearman’s correlation coefficient) or statistically differences were found. In other words, none of the blood types associated with better or worse performance on the psychomotor and neurofunctional tests (Figure 5A).

In contrast, for the Rh factor, a statistically significant correlation was observed in relation to the Clock Test (Spearman’s *r* = −0.53, *p* = 0.005**, Figure 5A). Figure 5B shows that Rh-negative (Rh-) subjects in the post-COVID-19 group performed worse on the Clock Test. A test of normality indicated that the data were not normally distributed (Rh-positive [Rh+]: *W* = 0.63, *p* < 0.0001****; Rh-: *W* = 0.92, *p* = 0.4633; ‘unknown’: *W* = 0.63, *p* = 0.0012**).

## Discussion

The present study divided the control and post-COVID-19 groups into three age strata (18–30, 31–45, and 46–64) to evaluate whether COVID-19 affects psychomotor and neurofunctional aspects differentially across age groups. These three age groups were defined based on existing scientific studies. The 18–30 age group is considered young adults. For instance, studies involving this age range include research in many areas, including, for example, type 2 diabetes (Wong *et al*., 2022) and suicide (Lipschitz, 1995). Several studies also focus on the 31–45 age group (middle-aged adults). Both specific age ranges – 18–30 and 31–45 age – were evaluated for differences in tooth shades (Bhaskaran & Sharma, 2022) and irritable bowel syndrome (Alrasheedi *et al*., 2025). In fact, there is also a study on COVID-19 that includes these two age ranges (Gonzalez-Andrade & Carrero, 2023). The 46–64 age group, considered older adults, has been studied, for example, in relation to the immunogenicity of an oral cholera vaccine (McCarty *et al*., 2019) and sickle cell disease (Xu *et al*., 2025). Thus, the three age strata (18–30, 31–45, and 46–64) represent standard divisions in scientific research.

According to psychomotor concepts, humans are considered predominantly ‘motor’ at birth. Through constant motor development, reflexive movements become voluntary and increasingly intentional and refined (Costallat, 1985). The disappearance of primitive reflexes, normalisation of muscle tone, acquisition of postural control, and development of cognition occur progressively. Eventually, performing more complex motor activities requires prior knowledge of the activity (Borges *et al*., 2010).

During ageing (retrogenesis), regressive manifestations of behaviour, motor, perceptual, and cognitive functions occur in reverse order to ontogenesis – from the cortex to the medulla, and from the most voluntary to the automatic functions (Fonseca, 1998). Evidence of functional retrogenesis – in terms of cognitive, neurological, and emotional functioning – coincides with the regression of myelination in frontal areas affected by Alzheimer’s disease (Borges *et al*., 2010). Fine Motor skills represent the last psychomotor function to fully mature, which justifies the use of psychomotor assessments to detect possible early senescence or neurodegeneration, based on the direct relationship between motor planning and cognition. In fact, neural damage caused by COVID-19 is similar to that observed in patients with neurodegenerative diseases or premature senescence (Borczuk & Yantiss, 2022).

The present study revealed that COVID-19 impaired performance in all three psychomotor tests, which included the Fine Motor Development Tests (Diadochokinesia, Puppets, Fan, and Paper) and Balance Tests (Immobility, Static Balance on One Foot, Feet in Line, and Persistence). Even 12 and 24 weeks after the initial assessment, COVID-19 continued to induce significant psychomotor impairments. The Fine Motor Development and Balance tests require not only postural tonic control but also movement planning and automatisation – ontogenetic acquisitions that develop later and are among the first to be affected in the process of retrogenesis. Notably, the most impaired age group – and the only one in which impairments persisted for at least 24 weeks – was the 31–45 age group. It is surprising that this group showed signs suggestive of premature ageing. The subsequent age group (46–64 years), which would typically be expected to show results more consistent with senescence, did not exhibit psychomotor impairments after 24 weeks. This reinforces a possible association between COVID-19 and features of early senescence.

There is growing evidence that various viruses, such as the human immunodeficiency virus (HIV) and SARS-CoV-2, have the capacity for neuroinfection and may contribute to premature ageing (Wu *et al*., 2020). These viruses have the potential to exacerbate age-related cognitive decline, as shown by neuroimaging studies revealing structural and functional changes in the brain (Cohen *et al*., 2015). In fact, there are reports of perceived premature ageing in patients who have recovered from COVID-19 (Duan *et al*., 2023). The mechanism that may explain post-COVID-19 premature ageing appears to be related to inflammation and oxidative stress triggered by the infection – both of which are known to play a key role in the biological ageing of cells (Cao *et al*., 2022).

In addition to the neurofunctional impairments associated with premature ageing, the post-COVID-19 group showed a significantly higher frequency of synkinesia at all three assessment time points. Synkinesias can be classified as: kinetic (involuntary movements of the contralateral limb), passive (mimicking the inducing movement), or tonic (involuntary stiffening of the passive limb during action). These unnecessary synergies typically lead to increased bodily fatigue (Núñez & León, 2017). Synkinesias are commonly observed in children under the age of seven, as they represent a transitional stage in manual coordination development, necessary for motor automatisation. However, when present in adults, synkinesias may indicate irreversible organic disorders (Costallat, 1985). Moreover, they are frequently found in patients with neurodegenerative diseases or those diagnosed with retrogenesis.

Regarding memory tests, the initial impairment observed in the 18–30 age group, which did not persist in the subsequent assessments (2 and 3), supports the importance of both favourable and unfavourable life experiences in regulating and maintaining emotional balance. Evidence suggests that resilience is required to cope with a range of adversities – from ongoing daily hassles to major life events – and that positive adaptation must be conceptually appropriate to the adversity encountered (Fletcher & Mustafa, 2013). Psychological resilience can be broadly defined as the capacity to sustain or recover psychological well-being during or after exposure to stressful disabling conditions (Serafini *et al*., 2020). Indeed, studies conducted before and during the COVID-19 pandemic showed that participants, on average, reported higher levels of perceived stress and anger during the pandemic compared with pre-pandemic levels. Pre-pandemic distress, secondary consequences of the pandemic (e.g., lifestyle and economic disruptions), and pre-existing social stressors were more consistently associated with young adults’ emotional distress than direct exposure to COVID-19-related health risks (Shanahan *et al*., 2022). The youngest age group in the present study exhibited stress levels not only due to the disease itself but also as a result of deprivations experienced both before and after the pandemic. From this perspective, adults and older adults may experience more prolonged periods of stress across their lifetimes, driven by factors such as limited prospects and abrupt changes in routine (e.g., job loss, relocation, or marital transitions). Could these cumulative life experiences contribute to greater stress resilience and stronger emotional regulation in older adults? The findings of this study appear to support the notion of enhanced stress resilience among older age groups.

It is important to note that the age-matched control group (18–30 years) was also experiencing confinement, economic hardship, and the abrupt changes brought about by the COVID-19 pandemic. However, unlike the post-COVID-19 group (of the same age), they performed within the expected range on the memory test. In fact, individuals who had a SARS-CoV-2 infection showed a significantly higher prevalence of depressed mood, somatic symptoms, and anxiety-like behaviour (Pedrosa *et al*., 2020). The combination of reduced resilience to stress and SARS-CoV-2 infection may have initially resulted in attention difficulties, which impacted memory test scores. In the follow-up assessments at 12 and 24 weeks, with improved outlook and adaptation – including the establishment of new routines – the situation began to normalise, and memory performance improved.

Sample loss was not considered a limitation of the present study, as it would have been impossible to prevent COVID-19 infection and reinfection. The study lasted for more than 18 months, so some sample loss was to be expected. Nevertheless, despite the sample attrition, statistically significant differences were found between the groups, with *p*-values as low as < 0.0001. Therefore, the sample losses did not compromise the results of the study.

When discussing key symptoms during the course of COVID-19 in patients who later developed neurofunctional impairments, correlations were observed between body pain and psychomotor test performance, coryza and memory test results, sore throat and clock test performance, and alteration of consciousness and verbal fluency scores. Notably, early studies on long COVID reported that 87% of participants experienced at least one symptom two months after infection, with body pain showing a prevalence of 27.3% (Carfi *et al*., 2020). A large cohort study conducted in Wuhan, China, found that six months after COVID-19 diagnosis, patients continued to present with fatigue or muscle weakness (52%) and sleep difficulties (26%) – symptoms now recognised as the most common manifestations of long COVID (Huang *et al*., 2023). Patients – particularly older individuals with comorbidities – often experience persistent dyspnoea, fatigue, body pain, and mental confusion for months following the acute phase of COVID-19 (Naeije & Caravita, 2021). Therefore, body pain has been proposed as a key symptom of COVID-19 associated with lasting impairments observed in psychomotor assessments. In fact, body pain in viral infections is commonly caused by an overreaction of the immune system, which leads to the release of large quantities of cytokines. SARS-CoV-2 is known to trigger a ‘cytokine storm’, involving the production and release of interleukin (IL)-6, IL-10, and tumour necrosis factor (TNF)-alpha. This cytokine storm can induce or exacerbate damage to various tissues, such as joints and muscles, thereby contributing to pain-related symptoms (Chen *et al*., 2020).

COVID-19 differs from other upper respiratory tract infections due to its high degree of chemosensory involvement (Sayin *et al*., 2023). However, this pronounced chemosensory involvement typically occurs in the absence of nasal obstruction or other inflammatory symptoms. In COVID-19, coryza (14%) and sore throat (15%) are less frequent than in other upper respiratory tract infections, such as Influenza A (70% for coryza and 45.5% for sore throat) and Influenza B (74% for coryza and 33% for sore throat) (Sayin *et al*., 2023). Patients with viral pneumonia had lower rates of coryza and sore throat than those without pneumonia (Iwata *et al*., 2022). Therefore, the presence or absence of coryza appears to be independent of the severity of the viral infection and may represent a useful traceable symptom.

The fact that both coryza and sore throat showed negative correlations with the Memory and Cock tests demonstrates that, although these are considered milder symptoms during the acute phase of COVID-19, they may play an important role in long COVID. Therefore, the presence of coryza and sore throat, along with body pain, should be considered key symptoms for screening for long COVID syndrome and associated psychomotor disturbances.

The Verbal Fluency Test (Nitrini *et al*., 1994) assesses the ability to recall words from a single category within a specified timeframe, providing a measure of comparative cognitive function. In the present study, a correlation was observed between lower scores on the Verbal Fluency Test and the occurrence of altered consciousness symptoms post-COVID-19. The pathophysiology of loss or alteration of consciousness during COVID-19 remains unclear (Fischer *et al*., 2022). Loss or alteration of consciousness due to other causes of brain injury – whether traumatic, anoxic, or cerebrovascular – is characterised by reduced neural connectivity, including functional connectivity as assessed by magnetic resonance imaging. Although alterations in consciousness occur in up to 20% of COVID-19 cases, they tend to return to a functional status over the course of months (Fischer *et al*., 2022). Therefore, the correlation identified in the present study may represent a transient finding, warranting further investigation and follow-up, including imaging studies that could help to clarify the underlying mechanisms.

When each medical care approach during COVID-19 was compared with each psychomotor and neurofunctional test, no significant correlations were found. In other words, the severity of the acute phase of the disease was not a determinant of better or worse performance on these tests. Thus, a patient who progressed to a severe stage of the disease may have recovered without psychomotor or neurofunctional sequelae, whereas a patient who experienced only mild COVID-19 symptoms and was never hospitalised may have developed marked psychomotor and neurofunctional sequelae. These findings highlight the importance of carefully monitoring symptoms during COVID-19. Symptoms such as body pain, coryza, and sore throat may appear minor or go unnoticed but should nonetheless serve as warning signs for patient follow-up.

There is no consensus in the literature about why some patients develop long COVID and experience sequelae, even though during the course of the infection they only had mild symptoms. Similarly, some patients who were admitted to an ICU never present long COVID symptoms. This pattern was also observed in psychomotor and neurofunctional aspects. Some authors explain this phenomenon by the unique characteristics of each patient’s immune system. For example, there is evidence that the levels of natural killer cells (Csordas *et al*., 2022), neutrophils (Wang *et al*., 2022), and monocytes (Chevrier *et al*., 2021), as well as the production of interferon, are hallmarks of severe COVID-19. Additionally, the Th1 cytokine endotype discriminates and predicts severe complications in COVID-19 (Hasegawa *et al*., 2022). An epigenetic (DNA methylation) mechanism may also be involved (Corley *et al*., 2021). Therefore, further studies of the immune system would help elucidate the distinct mechanisms between severe and mild COVID-19 in relation to psychomotor and neurofunctional changes.

No positive or negative correlation was found between psychomotor and neurofunctional performance and blood type in post-COVID-19 patients. In other words, no protective or detrimental effects were associated with any specific blood type. This investigation was undertaken because the literature on this topic remains highly divergent. Some studies suggest that certain blood types may confer a greater susceptibly to more severe viral outcomes. For example, a study in China involving 2173 COVID-19 patients reported that blood type A was associated with a higher risk of infection, whereas type O was associated with a lower risk (Zhao *et al*., 2021). In contrast, a similar study conducted in Saudi Arabia with 2302 COVID-19 patients found no association between blood type and disease severity or mortality (Halawani *et al*., 2024). Although the literature remains contradictory, the present findings support the hypothesis that blood type does not influence COVID-19 outcomes. Specifically, psychomotor and neurofunctional functions in the post-COVID-19 subjects were not affected by blood type.

Conversely, a negative correlation was found between the Rh- factor and performance on the Clock Test. The Clock Test, a component of the Mini-Mental State Examination, is one of the most widely used tools for dementia screening (Nitrini *et al*., 1994). This test evaluates spatial organisation, visuoconstructive abilities, and the organisation of graphic thinking. In healthy adults, a score of 10 is expected. Scores between 9 and 7 reflect progressively subtle errors, indicating mild spatial-executive cognitive dysfunction, whereas scores between 5 and 1 are considering warning signs of dementia when interpreted in conjunction with results from other cognitive assessments (Juby *et al*., 2002).

The Rh factor has been studied for similar reasons as blood type in relation to COVID-19. Evidence suggests that Rh+ patients are hospitalised less frequently (Turhan *et al*., 2022) and have lower mortality rates (Greco *et al*., 2021). Our findings support the hypothesis that Rh− individuals are more adversely affected by COVID-19. Specifically, Rh− status was associated with poorer psychomotor and neurofunctional performance following COVID-19 infection. Therefore, the Rh− factor may be considered a risk factor for COVID-19 outcomes.

The present study did not assess variables such as mood, stress, physical activity, dietary patterns, or socioeconomic context, which could potentially influence psychomotor and neurofunctional outcomes. While future research may address these factors, the current study included a control group matched for age distribution, with all participants from the same geographic and ethnic background. The evaluation of both control and post-COVID-19 groups was conducted concurrently. Therefore, these variables were not considered potential confounders in the present analysis.

The impact of sample size was evaluated for the four variables related to psychomotor and neurofunctional assessments. The results are presented in Supplementary Tables 1 and 2. Judgemental (or purposive) sampling achieved a very high statistical power (*η* ^2^ = 0.98, Eta-sq) for the ‘Psychomotor tests’ variable, supporting the number of participants included in each group in the present study. However, the sample size and power calculations for the remaining three variables did not indicate robust statistical power. Considering that the number of participants varied during the study due to COVID-19 positivity and the initiation of psychotropic medication – both exclusion criteria – the findings should be interpreted as providing preliminary support for further, more extensive research in this field.

Sample size and statistical power were calculated using age-stratified subgroup data. The results indicated test power ≥ 80% (based on η^2^ and RMSSE), demonstrating a strong ability of the statistical tests to detect existing effects and thereby minimising the likelihood of Type II errors (i.e., failure to detect a true effect). Despite the small sample size obtained through a non-probability sampling method – namely judgemental (or purposive) sampling – the data demonstrated good sensitivity. Consequently, the present findings may contribute to clarifying COVID-19 – related outcome, including persistent psychomotor and neurofunctional sequelae. Sample size calculations are provided in the Supplementary Material.

Taken as a whole, the findings indicate that COVID-19 has both indirect and direct effects on the central nervous system, potentially leading to early neuronal senescence, long COVID symptoms, and psychomotor and neurofunctional sequelae for at least 24 weeks following the resolution of the acute disease. The present assessment protocol and the screening of key symptoms (such as body pain, coryza, and sore throat) may serve as tools to identify patients who should be more closely monitored for persistent long COVID symptoms and potential need for rehabilitation.

The medical and paramedical community must be aware of a potential rise in neurodegenerative diseases, particularly among individuals aged 31–45, the group that showed the most significant and persistent psychomotor impairments post-COVID-19. It is essential that governments prioritise public policies for screening, monitoring, and developing rehabilitation protocols, as sequelae in this age group could affect both the healthcare system and the economy, given that they constitute the majority of the economically active population (IBGE, 2023).

In conclusion, the present study demonstrated that COVID-19 induced psychomotor and neurofunctional sequelae lasting at least 24 weeks after the acute phase of the disease. These impairments included deficits in fine motor development, balance, and Mini-Mental State Examination performance, as well as the induction of synkinesis. Moreover, different age groups exhibited distinct patterns: psychomotor impairments were more pronounced and persisted longer in the 31–45 age group, whereas individuals aged 18–30 years showed greater memory impairments. A negative correlation was observed between psychomotor performance and body pain, coryza, and sore throat during COVID-19, which were therefore considered key symptoms. However, no correlation was found between the level of medical care and psychomotor performance, highlighting that patients with mild symptoms during the acute phase of COVID-19 also require medical attention and monitoring. Finally, the Rh− blood type was suggested as a potential indicator of psychomotor sequelae after COVID-19.

## Supporting information

Chiminazzo et al. supplementary material Chiminazzo et al. supplementary material

## Supplementary material

The supplementary material for this article can be found at https://doi.org/10.1017/neu.2026.10067.
