# Supplementary material for: Psychomotor and neurofunctional sequelae after COVID-19
Source: Acta Neuropsychiatr. 2026 Mar 5;38:e25. doi: 10.1017/neu.2026.10067 (PMC13130310; doi:10.1017/neu.2026.10067)
Supplement: Chiminazzo et al. supplementary material [file S0924270826100672sup001.pdf]

Psychomotor and neurofunctional sequelae after COVID-19 – **Supplementary Tables**

Supp. Table 1. Normality tests performed for the Psychomotor Tests, Memory Test, Verbal Fluency Test, Clock Test, and Synkinesis Frequency. Normality of the data distribution was verified using the Shapiro-Wilk (W) or Kolmogorov-Smirnov (KS) tests, depending on the sample size. All assessments – Evaluations (EV) 1 (initial), 2 (after 12 weeks), and 3 (after 24 weeks) included comparisons of the post-COVID-19 group with the respective control groups (n=30/30 on EV1, 20/30 on EV2, and 13/28 on EV3 for the control and post-COVID-19 groups, respectively

		**Evaluation 1**		**Evaluation 2**		**Evaluation 3**	
**Test / Group**	**Normality test**	**control**	**post-COVID-19**	**control**	**post-COVID-19**	**control**	**post-COVID-19**
**Psychomotor Tests**	non-normal distribution	W=0.97, *p*=0.4294	W=0.89, *p*=0.0060**	W=0.95, *p*=0.3287	W=0.96, *p*=0.3152	W=0.93, *p*=0.3482	W=0.96, *p*=0.3702
**Memory Test**	non-normal distribution	W=0.86, *p*=0.0008***	W=0.91, *p*=0.0128*	W=0.80, *p*=0.0008***	W=0.89, *p*=0.0054**	W=0.78, *p*=0.0038**	W=0.94, *p*=0.0987
**Verbal Fluency Test**	non-normal distribution	W=0.96, *p*=0.3383	W=0.97, *p*=0.5198	W=0.97, *p*=0.7182	W=0.90, *p*=0.0086**	W=0.92, *p*=0.2586	W=0.92, *p*=0.0447*
**Clock Test**	non-normal distribution	KS=0.53, *p*<0.0001****	KS=0.31, *p*<0.0001****	KS=1.00, *p*<0.0001****	KS=0.42, *p*<0.0001****	KS=1.00, *p*<0.0001****	KS=0.48, *p*<0.0001****
**Synkinesis Frequency**	non-normal distribution	W=0.90, *p*=0.0078**	W=0.50, *p*<0.0001****	W=0.91, *p*=0.0618	W=0.52, *p*<0.0001****	W=0.93, *p*=0.3559	W=0.67, *p*<0.0001****

**p*<0.05, ***p*<0.01, ****p*<0.001, and *****p*<0.0001

Supp. Table 2. Normality tests performed for the Psychomotor Tests, Memory Test, and Verbal Fluency Test evaluated among the three age groups. Normality of the data distribution was verified using the Shapiro-Wilk (W) Test. All assessments – Evaluations (EV) 1 (initial), 2 (after 12 weeks), and 3 (after 24 weeks) included comparisons of the post-COVID-19 group with the respective control groups (n=30/30 on EV1, 20/30 on EV2, and 13/28 on EV3 for the control and post-COVID-19 groups, respectively

		**18–30 years old**		**31–45 years old**		**46–64 years old**	
**Test, Evaluation / Group**	**Normality test**	**control**	**post-COVID-19**	**control**	**post-COVID-19**	**control**	**post-COVID-19**
**Psychomotor Tests, Evaluation 1**	normal distribution	W=0.83, *p*=0.1012	W=0.96, *p*=0.8221	W=0.94, *p*=0.4795	W=0.88, *p*=0.0908	W=0.98, *p*=0.9598	W=0.93, *p*=0.3699
**Psychomotor Tests, Evaluation 2**	normal distribution	W=0.92, *p*=0.5300	W=0.98, *p*=0.9471	W=0.95, *p*=0.6641	W=0.96, *p*=0.7162	W=0.92, *p*=0.5241	W=0.95, *p*=0.6523
**Psychomotor Tests, Evaluation 3**	normal distribution	W=0.94, *p*=0.6499	W=0.92, *p*=0.5027	W=0.94, *p*=0.6646	W=0.93, *p*=0.3419	W=0.92, *p*=0.5381	W=0.86, *p*=0.0704
**Memory Test, Evaluation 1**	non-normal distribution	W=0.64, *p*=0.0014**	W=0.98, *p*=0.9600	W=0.70, *p*=0.0005***	W=0.81, *p*=0.0097**	W=0.86, *p*=0.0655	W=0.89, *p*=0.1259
**Memory Test, Evaluation 2**	non-normal distribution	W=0.87, *p*=0.2117	W=0.91, *p*=0.4150	W=0.57, *p*<0.0001****	W=0.87, *p*=0.0551	W=0.82, *p*=0.0911	W=0.82, *p*=0.0164*
**Memory Test, Evaluation 3**	non-normal distribution	W=0.73, *p*=0.0239*	W=0.90, *p*=0.3888	W=0.68, *p*=0.0065**	W=0.96, *p*=0.7903	W=0.63, *p*=0.0012**	W=0.88, *p*=0.1239
**Verbal Fluency Test, Evaluation 1**	normal distribution	W=0.95, *p*=0.7366	W=0.80, *p*=0.0605	W=0.89, *p*=0.0923	W=0.97, *p*=0.9133	W=0.92, *p*=0.2957	W=0.97, *p*=0.8375
**Verbal Fluency Test, Evaluation 2**	non-normal distribution	W=0.92, *p*=0.5141	W=0.77, *p*=0.0306*	W=0.94, *p*=0.6445	W=0.87, *p*=0.0573	W=0.90, *p*=0.3888	W=0.94, *p*=0.4699
**Verbal Fluency Test, Evaluation 3**	non-normal distribution	W=0.75, *p*=0.0409*	W=0.83, *p*=0.1133	W=0.92, *p*=0.5509	W=0.96, *p*=0.8196	W=0.86, *p*=0.2469	W=0.87, *p*=0.0965

*$p<0.05$, **$p<0.01$, ***$p<0.001$, and ****$p<0.0001$
